# Interprofessional collaborative learning in the workplace: a qualitative study at a non-governmental organisation in Durban, South Africa

**DOI:** 10.1186/s12909-020-02264-5

**Published:** 2020-10-06

**Authors:** Sarentha Chetty, Varsha Bangalee, Petra Brysiewicz

**Affiliations:** 1 Discipline of Pharmaceutical Sciences, School of Health Sciences, Westville Campus, University of KwaZulu-Natal, Durban, South Africa; 2 School of Nursing & Public Health, College of Health Sciences, University of KwaZulu-Natal, Durban, South Africa

**Keywords:** Interprofessional, Education, Healthcare, Collaboration, Nursing, Medicine, Pharmacist, Nursing, Allied health

## Abstract

**Background:**

The rapid progression of diseases and the complex, changing landscape of healthcare has increased the awareness that interprofessional collaboration is essential in ensuring safe and effective healthcare delivery. However, to develop a “collaborative practice-ready” workforce, organisations need to invest in the application of alternative approaches to the training of healthcare professionals.

**Purpose of the study:**

To describe the perceptions of healthcare professionals attending an HIV interprofessional collaborative initiative at a non-governmental organization research site in South Africa and to provide suggestions regarding the improvement of this educational programme.

**Methods:**

Focus group discussions (December 2018 to January 2019), were conducted on a purposeful sample (*N* = 21) consisting of healthcare professionals (clinicians, pharmacists, pharmacy assistants, and nurses), and clinical trial staff (recruiters, administrators, QC officers, psychologists, counsellors) based at a research site, who were invited to attend a continuing medical education initiative on the pathogenesis and treatment of HIV. Qualitative content analysis was carried out to identify meaning units, which were then condensed and labelled with a code. This was further grouped to form categories.

**Results:**

Five categories emerged: learning something new, acquiring from each other, promoting company culture, needing company buy-in and teaching methods matter. Interprofessional collaborative learning improved technical capacity, work relationships and company culture. The diversity in learning needs of the different professionals requires a structuring of a curriculum to meet the needs of all. The success of this initiative requires company buy-in/investment and recognition from leaders and higher management with regards to time and resources. Suggestions for improvement included: formalizing the training, introducing more lectures and pitching each topic at different levels i.e. basic, intermediate or advanced, thus ensuring maximum benefit for all.

**Conclusion:**

Inter-professional learning was perceived as highly valuable. This initiative has the potential to develop further but requires resources and company buy-in. All staff working (clinical and non-clinical) at the NGO site were represented in the interviews, thus ensuring a richer understanding of all perspectives relevant to the study site. The small sample size confined to a single research site, however, prevents these findings from being generalized and limits the applicability of its findings.

## Background

The World Health Organization (WHO) recognises the importance of, and strongly encourages the incorporation of interprofessional education efforts in all healthcare professional training programs. The rationale for this being that patient and population outcomes are improved through multidisciplinary and collaborative care [1]. Interprofessional collaboration, at its core, occurs when two or more professions work together to achieve common goals and is often used as a vehicle for solving a variety of problems and complex issues for the benefit of the patient [2]. Interprofessional education (IPE) “occurs when students from two or more professions learn about, from, and with each other” [3], p13. There is much evidence that interprofessional teams have the capability of enhancing patient care [4–9]. The WHO encourages interprofessional education, especially in the field of medicine, as it promotes interaction and learning together of essential skills to solve a health-related problem [6]. In order to create a genuinely interprofessional education experience, efforts are required to ensure that such interaction purposefully provides integration and collaboration among the disciplines, whether in an educational or practice environment [10, 11]. There have been several attempts to achieve interprofessional education at an undergraduate level [12–14]; however, to sustain these principles, interprofessional education needs to transcend beyond undergraduate training and into the workplace. Acknowledging the importance of training a “collaborative practice-ready workforce” [3], p11, thus requires the application of an alternate approach to the training of healthcare professionals. One possible method is to explore interprofessional collaborative education through continuing medical education (CME) initiatives.

The importance of CME or in-service training for healthcare professionals following the completion of basic training has long been recognised for its ability to maintain and update competence, as well as to learn about new and developing areas in the field [15, 16]. CME activities may include workshops, seminars, clinical audits, formal lectures, written publications, online programs, audio, video, or other electronic media. Studies reveal that people learn through repetition and that skills are easily educed if regularly revised [15]. It thus stands to reason that healthcare professionals, the foundation of our healthcare system, also require a structured process of continuous training, exposure, learning, and improvement in order to apply their skills and knowledge correctly [15].

An interprofessional collaborative CME initiative was conducted at a non-governmental organisation (NGO) specializing in Human Immunodeficiency Virus (HIV) and tuberculosis (TB) research. Being a newly implemented initiative at the research site, it was essential to obtain participants’ perceptions of interprofessional collaborative education through the CME initiative to determine the perceived value of this type of educational strategy.

### Purpose of the study

To describe the perceptions of healthcare professionals attending an HIV interprofessional CME collaborative learning initiative and to provide suggestions regarding the improvement of this educational programme.

### Research objectives

The research objectives were to:

- Describe the perceptions of participants attending an HIV interprofessional collaborative CME initiative
- Provide suggestions regarding the improvement of this interprofessional collaborative CME initiative

## Methods

### Study approach

This was a qualitative content analysis [17] study using focus group discussion (FGDs).

### Study setting

The NGO is involved in basic research as well as several HIV and TB clinical trials. The research site runs HIV prevention studies (vaccines and pre-exposure prophylaxis) and treatment studies (HIV, TB, and HIV-TB co-infection). In 2018, the NGO employed a total of 77 staff. This included 8 clinicians, 4 pharmacists, 2 pharmacy assistants, 19 nurses, 26 clinical trial staff and 18 site operational staff.

A monthly CME activity was initiated to serve as a refresher and update; for some of the members of staff, it presented a first-time introduction to the HIV theory. The CME activities (open to all) provided an overview of various HIV topics of concern to the staff employed at the research site. Some of the topics included: Introduction to HIV (epidemiology, virology, and pathogenesis), HIV treatments, HIV and TB co-treatment, HIV standard treatment guidelines, HIV case studies, HIV drug resistance, HIV gender issues, pre-exposure prophylaxis (PrEP), HIV vaccines and the role of nutrition and food hygiene in HIV.

Each lecture was supplemented with multiple-choice questions (MCQs), case studies, and group exercises, which were used to increase comprehension, promote active learning and encourage application to real-life situations. Although all staff were invited to attend the CME activities, all did not consistently attend the lectures. There was, however, representation from the clinicians, pharmacy, nurses and clinical trial staff.

### Research participants

Purposive sampling was used to recruit participants that attended the CME activities.

Inclusion criteria for all participants were as follows:

i.) staff that were employed at the research site and
ii.) had attended one/more CME activities and
iii.) participants who were healthcare professionals (had current registration with their respective healthcare professional governing body).

Exclusion criteria included all staff who were employed at the research site but had not attended any of the CME activities.

### Data collection process

All FGDs took place at the research site during normal working hours. The study was conducted over 2 months (December 2018 to January 2019) on completion of the HIV CME activities for 2018.

Participants that attended the CME were recruited from the attendance registers of each session.

FGDs were chosen as the data collection method as it encourages participation from those participants who may feel they have nothing to contribute or may feel intimidated if interviewed alone. FGDs also attempts to create an environment in which participants feel free to communicate with one another and discuss [18, 19].

The participants were adult learners from different educational and professional backgrounds, thus to ensure the most conducive atmosphere the participants were divided into four separate FGDs with a maximum of 10 participants. This was based on professional backgrounds and research tasks at the site:

- Group 1: Clinicians – registered with the South African Medical Association and Health Professions Council of South Africa
- Group 2: Nurses- registered with the South African Nursing Council
- Group 3: Pharmacists and pharmacist assistants – registered with South African Pharmacy Council
- Group 4: Clinical trial staff - recruiters, administrators, counsellors, qualitative control (QC) officers, recruiters, research assistants, and study coordinators

Demographic data (Supplementary, S1) was requested from the participants before starting the FGD. The FGD commenced after providing an introduction and explanation of the purpose of the study and the ground rules by the researcher (SC) who was known to the participants. A request was also made for permission to record the session.

The FGD consisted of four questions (Supplementary, S2).

The first question “please tell me about your experience in attending these CME activities” was posed to ascertain the general response of the group to the CME activity and to extract general viewpoints on these CME activities and if/how it facilitated learning. The follow-up questions were used to investigate the advantages, disadvantages/challenges and possible suggestions for improving the activity. The question “What are the advantages of attending an HIV CME lecture” was aimed to investigate if the participants found it beneficial to learn together with different health professionals, and clinical trial support staff, as well as staff from across different studies (treatment and prevention). The question “What are the challenges/disadvantages to attending an HIV CME lecture……” was asked to determine if there were any disadvantages of attending the CME activity with other professionals and staff from different research clinics. The last question, “What are the possible suggestions for improvement of the CME activity?” was used to determine how the CME activity could be improved in order to structure future sessions.

### Data analysis

The FGDs were transcribed verbatim [19], and manifest content analysis was used to analyse the data [17, 20]. After reading and re-reading transcripts, meaning units were identified, condensed, and labelled with a code. Codes were then grouped to form categories [17, 20]. Please see Supplementary, S3 for examples of content analysis coding and categorization.

### Rigor

All efforts to maintain academic rigour were made to meet the required qualitative criteria of trustworthiness which includes credibility, transferability, dependability and confirmability [21, 22].

The credibility of the study was ensured as the researcher (SC), an employee at the NGO, had familiarity with the culture of the study setting. Participants were requested to be honest in their responses, reassuring them that there were no “correct” responses to the questions. Each participant who was approached was further allowed to refuse participation in the project to ensure that the data collection sessions only involved people who were genuinely willing to partake and offer data freely. Data analysis was conducted by the entire research team (comprised of experienced qualitative researchers) and involved individual analysis, group discussion and interrogation of the emerging categories. Triangulation of data sources was used to ensure the categories and conclusions were confirmed by the different professional groups. Transferability was assured by providing a detailed description of the context of the study to allow for sufficient detail to be provided for the reader. A detailed description of the research process was also provided to assist with dependability, together with a reflective evaluation of the project. Confirmability was assured through the keeping of records and transcripts of each FGD.

### Ethics

Ethical approval was granted from the Humanities and Social Sciences Research Ethics Committee of the University of KwaZulu-Natal, South Africa (Protocol Reference number HSS/2093/018), as well as permission from the NGO to carry out the study at the research site. Participation in the study was voluntary with no linked identifiers. Participants had the right to decline participation or to withdraw from the study at any time. Prior written consent was required before the participants chose to participate in the study. Permission to record FGDs was also obtained. To ensure anonymity and confidentiality, the participants were assigned codes.

## Results

### Descriptive characteristics

Twenty-one staff members participated in this study. The composition of participants were as follows: 4 research clinicians (19%), 4 pharmacy staff (19%), 6 nurses (29%), and 7 clinical trial support staff (33%). There were 2 males and 19 females. Four aged between 20 and 30 yrs. (19%), 8 between 30 and 40 yrs. (38%), and 9 over 40 yrs. (43%).

Five categories emerged from the data: learning something new, acquiring from each other, promoting company culture, needing company buy-in and teaching methods matter.

### Learning something new

Participants described how the CME activities were beneficial in that it provided an ideal forum for building upon and updating individual knowledge on HIV, new advancements in the field and HIV guidelines, which are always changing. One of the participants commented:

E*verything is changing so quickly we need to learn the newer ways… the better ways of doing things.* (Nurse, P21)

The clinicians and pharmacists, in particular, explained that in addition to being a refresher, the CMEs were an excellent way to educate the rest of their research team by providing access to information that they would not have necessarily been exposed to.

*For my team getting insight into information* [area] *that we don’t work in on a daily basis.* (Clinician, P1)

For some attendees, the CME activities stimulated reflection and identification of individual knowledge gaps. Following this, they were then motivated to pursue further learning beyond the lectures on areas of interest. A participant explained:

*Something that was just touched on in the lecture and you wanted to remember more about or get more information on ….[we] went back and looked for it.* (Pharmacy staff, P5)

*Continuing of thinking even after the presentations even at team meetings as well there would have been stimulated discussion around the subject, which is good because I don’t think people would have thought otherwise if the lectures had not stimulated that thinking process*. (Clinician, P1)

The CME activities prompted non-clinical staff (referred to as junior staff) to seek further clarity on unfamiliar concepts from clinicians, thus additionally encouraging communication and further engagement between staff members.

*I found that after the lectures I was actually asked some questions from the junior staff members that they probably would not have asked if they did not attend. I think it kind of stimulated people to think*. (Clinician, P3)

Many of the participants explained that the content was relevant to their practice and exposure to this information built capacity. The clinical trial staff, some of whom had very little theoretical background on HIV voiced that the CME activities helped build their confidence to converse about HIV topics with other healthcare professionals and with patients.

*…this type of training gives us confidence when we talk about HIV.* (Clinical trial staff, P13)

The participants suggested that the CME activity could be more useful and informative for all if they were given time to prepare beforehand. A participant explained:

*..some time to prepare for that topic and forming some questions. In that* [so that] *we don’t see the information for the first time when it is presented.* (Clinical trial staff, P11)

*I felt I was not prepared for the lecture as a result I don’t know if I gained much……. ..I need maybe to have something to read or something to read before or take home and revise.* (Nurse, P16)

The participants wanted to play an active role in contributing to the development of CME activities and would prefer more input into their training needs.

*I know there is time constraints and workloads but something to consider is if we get the different teams to present a topic. Maybe prevention one day. Maybe even if ….maybe not viable because of the workspace but mix the groups from prevention and treatment and they present.* (Clinician, P2)

*..could ask them what topics that they are more interested in learning about, what people have a large gap in.* (Pharmacy staff, P7)

### Acquiring from each other

The staff were all in agreement that the interprofessional collaborative learning initiative was valuable and provided an opportunity to learn from each other. It exposed them to different levels of knowledge not just from the different professionals but also from the different professional experiences that they each had.

*It is also nice because you have different people from different sectors. So, you have your pharmacy, your lab staff, you have counsellors, recruiters and you have input from everybody.* (Clinical trial staff, P9)

*Important to learn from experience…. learning from experiences as opposed to learning just directly even just trying to read because most of us know as well that within medicine a lot of it comes from personal experience.* (Clinician, P4)

Clinicians described how learning with other professional groups forced them to view the topic from a different viewpoint.

*…advantageous and the reasons were again we picked up things from other people which helped in how you would manage care*. (Clinician, P4)

In this regard they acknowledged that patients may be more comfortable to divulge certain information to the other healthcare professionals and that this could be crucial to the patient’s well-being.

[We] *tend to think in a particular way….very scientifically or very matter of fact… but some other members brought in different aspects that maybe you would overlook which is important like the social factors.* (Clinicians, P3)

*Often in our interactions with patients, their interactions with a doctor may be very different from their interaction with a counsellor …. maybe they may not ask us certain questions or certain things may not come forth in your interactions with them. There is a lot to learn from their* [other professionals] *interaction with the participants.* (Clinician, P4)

The group discussions that ensued during the sessions helped attendees put the knowledge gained from the CME activities into perspective.

*Refreshing to get other professionals’ perspectives because you know that leads to misconceptions, misunderstandings or just* [a] *general take on the topic and getting the information out there loud and clear*. (Clinician, P1)

Interprofessional collaborative learning provided participants with greater role clarification and responsibilities of each healthcare professional. They began to identify their role in the healthcare team and how each member’s role contributes to the overall functioning of the site thus improving individual and the overall workplace familiarity.

*Makes it much better and gives more direction when everyone is clear on what everyone is meant to do and what their roles are.* (Clinician, P1)

*Staff from different disciplines they may speak about something that we have no knowledge about, and if they comment on it, or give you an explanation then you are learning more about that.* (Clinical trial staff, P12)

The participants did voice that one challenge to interprofessional collaborative learning was that it could be intimidating. Certain participants expressed that they were not confident in their background knowledge hence were apprehensive about sharing or expressing their views because there were other, more knowledgeable participants present. They were also less likely to ask or answer questions for fear of being incorrect.

*Sometimes if you do these trainings with the doctors… some people maybe they will ask some difficult questions and some of the times you are scared to answer or they* [we] *are embarrassed to ask these questions, but if they do ask you find this information so it is very important for you to attend because the person may be asking the question when you* [the presenter] *are explaining you realise I needed that information.* (Clinical trial staff, P10)

*…different groups and you are all at different levels and that if you voice your opinion it won’t be the right opinion.* (Nurses, P18)

It was found, however, that as the interactions increased, participants felt more comfortable to contribute to the discussions that ensued.

*After the first lecture, people became familiar with each other and they did open up and relax and communicate better.* (Nurses, P21)

The pharmacy staff also voiced that having a speaker from the organisation that they were familiar with as opposed to an external speaker made it less intimidating and encouraged people to engage more.

*A peer giving the lecture is that you are more comfortable. Sometimes when you have external speakers they may seem a little intimidating to approach.* (Pharmacy staff, P7)

Some participants felt that due to the heterogenicity of the group there would be different levels of understanding which could sometimes present a problem.

*..certain professionals will understand certain things at certain levels.* (Nurses, P17)

Some of the senior medical staff, however, felt that even though the content of some lectures may be beyond the scope of certain participants it was still important that all members of the research team have some understanding of HIV.

*Certain lectures may be beyond the level of some people. But in the space we work in when we* [are] *exposed to research protocols these professions are exposed to* [a] *certain level of science or clinical research. I think the thinking should be there. We don’t expect them to understand everything but there should be a certain level of thinking that we do expect from an administrator for example.* (Clinician, P1)

The nurses suggested that the information should be broken down further to a more fundamental level to facilitate understanding of complex concepts. They also felt more lectures from a foundational to a more advanced level would promote understanding of complex topics, for attendees without clinical backgrounds.

*A lot of information on certain lectures and concepts, if it could be broken down more with certain things. Complex subjects… more lectures so the content is spread out.* (Nurse, P21)

Another suggestion was to grade/label the lecture according to its level: basic, intermediate and advanced. In this way, participants would be aware in advance of the level at which the lecture would be pitched, thus allowing them the choice as to which activities would be beneficial to them. This would prevent attendees without the relevant clinical background from losing interest and allow for more in-depth teaching of the subject for those with clinical backgrounds.

*For me, one challenge – will be for the presenter must have been to find the right level to present at because you have a whole spectrum of people and different levels and you need to engage each one and that is a challenge as you don’t want it to go over some people’s heads and you don’t want it at too low a low level so that they are not stimulated.* (Clinician, P3)

*..maybe splitting the lectures into basic and intermediate and so on. So basic for those that want the basic info and having intermediate and advanced for those who are at a different level or specifically interested in that topic or more clinical nature of the content.* (Pharmacy staff, P5)

An additional suggestion for improving interprofessional collaborative learning included using more small-group learning methods, as participants enjoyed working in smaller interdisciplinary groups to solve problems.

*So, it was very nice during the case studies to sit together and work and discuss and say ok we do this and someone else would say ok we do that and come together to find a common solution. So, it was good to interact with the other nurses and clinicians in that sense.* (Nurses, P21)

### Promoting company culture

Learning together promoted company culture/community, in that it improved knowledge about the company, the different clinical trials and research carried out at the site. One member of the clinical trial group mentioned:

*..there were a lot of studies that were going through* [the NGO] *where some of us were not involved* [in] *and we had no information about.* (Clinical trial staff, P13)

It additionally provided a comfortable space for discussion and communication. It fostered relationships and strengthened communication links not only interdisciplinary but also across and within research teams.

*Having these series of lectures and learning and laughing together breaks a lot of ice between the professions, and I think that I will feel more comfortable and I think a lot of the other staff will feel more comfortable to discuss any number of issues now because we have that rapport built.* (Clinician, P2)

*It was nice to see who works at* [the] *site. We don’t often get the chance to mix as a team and your lectures gave us the opportunity to put names and faces together just generally which is good.* (Clinician, P1)

*It gave us an opportunity to learn from other teams as well as share our knowledge from the space in which we work within. It gave a different perspective on how we do things as it is not always the same. It was just a great platform to share information.* (Clinician, P1)

Each of the research teams felt that they often worked in silos and that interprofessional collaborative learning broke down these barriers and facilitated integration. Several participants commented:

*We work very much in isolation, so it is a great point to come together at scheduled points and share knowledge.* (Clinician, P4)

*..these separate teams’ treatment, prevention or what were microbicides created sort of silos within an organization.* (Pharmacy staff, P5)

### Needing company buy-in

The participants unanimously felt that the CME activity required greater organisational support. They felt that this would be advantageous to staff and the organisation. For staff, gaining knowledge about all clinical trials carried out within the site enabled cross-study movement and job versatility. One participant explained:

*As staff working for such an organization, it* [we] *should have an understanding across the board and to me where there are people from treatment attended lectures on vaccines at a level which would have them understand if they had to move into that field, so that to me was really useful. (Pharmacy staff, P5)*

For clinicians and pharmacists continuing professional development (CPD) is a compulsory requirement for registration with their professional bodies. They felt that formalised training within the organisation would offer the convenience of meeting their professional CPD requirements. Finding time to read up on certain topics after hours was difficult. An in-house CME was beneficial in that it allowed them to learn whilst on site.

*CPD has actually become compulsory now and the employer should be assisting in meeting the CPD requirements and this is one simple way in which this can be met.* [A] *really easy way of doing it. It is all in-house basically.* (Pharmacy staff, P7)

*It was beneficial as in your day to day job you don’t have much time to read around subjects that are not directly related to your job.* (Clinician, P3)

An operational barrier that was cited as a significant challenge across all FGDs was the lack of time to attend CMEs. It was noted that in order for the CME to go forward, the organisation should acknowledge the capacity-building potential and convenience of an in-house CME. CMEs required dedicated and protected time for all interested staff to attend.

*Like journal clubs the research space in which we are in, it’s mandatory that we should improve learning, and continuing education is one of the main things that we should be doing. So if one of the things that* [we] *should get out of this initial lectures, that were held last year, is that we should move it forward, that it should happen in the future again, but in a more structured manner, we have buy-in from the management and leadership both at the site and maybe at director level as well so that sites have at least an hour a week or at least an hour every two weeks where clinics close at maybe 3 pm on whichever weekday and we have the ability to attend such a CME, be it this sort of lecture or any other type of workshop.* (Pharmacy staff, P5)

*I would have a day set aside for training because this is very important. It is a very important aspect of our work. So, if it is that important let us not put it like it as an afterthought.* (Nurses, P18)

Participants often complained that the lack of adequate staff in clinics to support co-learners and the pressure to be clinically productive stifled their ability to attend all CMEs on time or at all. A few participants voiced that due to busy clinics they were unable to attend and perhaps missed out on the basic level lectures and therefore found it more difficult to understand the lectures that followed. Others expressed that due to clinic pressures they were sometimes unable to stay for the full lecture and therefore unable to get the full benefit of the CME activity.

*Just making the time to attend and putting the clinic on hold and getting all the team members to attend. That was the biggest challenge for me.* (Clinician, P1).

*I ended up attending only one lecture because the clinic would be packed.* (Nurse, P20)

*It is very important to attend every lecture. I think time was the factor and why we all did not benefit from the full series.* (Clinical trial staff, P9)

[We] *wish* [to be] *able* [to] *attend all so there is a flow or if you stayed for the whole lecture, but if you missed two sessions or come in late then it would be difficult.* (Clinical trial staff, P15).

*Sometimes you have half in the room* [then] *you see some other people going out, busy with the clinic floor. They have to leave the session and go attend to the participant so you don’t have the time.* (Clinical trial staff, P14)

They also felt that there should be organisational support with regards to resources required to conduct CMEs such as the provision of notes and venues. They felt maybe IT assistance and maintenance with regards to a portal or SharePoint in which to view the lectures would be beneficial.

*Going forward if they have to formalize this type of CME maybe they could create a portal or use SharePoint where people who can’t attend can view it. Or like* [in the NGO weekly e-mail communication] *they will announce this talk so that everybody knows the time, date, venue, topic* etc. *and then make the material available to all on SharePoint or something like that so we have access before and after.* (Pharmacy staff, P6)

*If it could be possible that this information could be available maybe in the public domain.* (Clinical trial staff, P13)

One of the staff members felt that if it was formalized it would be a good way to promote personal development by building it into their key performance deliverables.

*If they could build it into our deliverables or something to say attend this type of talks or lectures than this is another way to make it more so.* (Pharmacy staff, P6)

### Teaching methods matter

All participants voiced that learning was greatly influenced by the teaching methods employed in delivering the CME activity. Participants felt that active learning techniques which included experiential learning through case-studies based on real-life scenarios and videos had promoted better understanding and knowledge retention. Participants were able to see the value of its application in educating their own communities, even beyond the research site. It promoted authentic learning.

*I liked the case studies quite a lot. Even though it was clinical it made you think out of your own whatever you can do, whatever your qualification is.* (Clinical trial staff, P12)

*We actually learnt. You know the case studies were really very helpful. ……….because some of these things are things we have seen, so even now in our own communities, we now know how to advise people from what we have learnt.* (Nurse, P18)

The clinicians felt that case studies gave other members of the team a better understanding of why specific processes were carried out in a certain way.

*Showed other professions in the team why certain management processes were occurring… need to think analytically because theoretical knowledge does not always make us think analytically so presenting practical cases kind of opens up those doors.* (Clinician, P1*)*

In lessons where participants were asked to prepare a particular section, it stimulated discussion on the subject between healthcare professionals and each gained from the expertise of the other. It further encouraged interprofessional collaborative learning, as attendees gained from the various expertise that emerged from discussions stimulated by the case-studies.

*That stimulated learning between me and the other person who had to prepare. That particular person hadn’t worked in the area of HIV treatment before and so she had a lot of questions.* (Clinician, P3)

*With her experience in counselling and my previous experience in the HIV treatment sector, together we were able to mesh our knowledge and it stimulated learning.* (Clinician, P3)

The interactive and varied nature of the activities appealed to the different learning styles of the participants as some learners are visual and others auditory.

*Videos, case reports it breaks the monotony. When you use different styles somehow the knowledge stays better.* (Clinician, P4)

*….everyone learns things in different ways. Some need to read, some to hear, some to discuss, so it was good.* (Nurses, P21)

As the CME activities were not structured didactically, the level of open engagement between attendees and the presenter provided a conducive environment for promoting interaction and learning.

*I also thought it wasn’t done in a very formal manner. A lot of work was done where it was very interactive, and questions were asked. (*Pharmacy staff, P5)

## Discussion

Attendees’ perceptions about an HIV interprofessional collaborative CME learning initiative were invaluable in determining the benefits, appropriateness, and challenges associated with instituting an interprofessional collaborative education learning platform. It additionally provided an arena for suggesting improvements, which are vital to the optimal structuring of future CME activities. Five categories emerged from the data: learning something new, acquiring from each other, promoting company culture, needing company buy-in and teaching methods matter.

Participants across all groups, regardless of the professional group or qualification, agreed that the CME covered relevant information and, in addition to being a refresher, also exposed them to new knowledge. To healthcare professionals, the concept of continuing professional development (CPD) is not new. The dynamic nature of the medical field, with its continually evolving technology, advances, and innovations, always requires healthcare professionals to continue their education beyond the undergraduate level to maintain exceptional patient care and advance their careers [23, 24]. CME allows medical professionals to identify and fill the gap in their knowledge and so develop and maintain competence. It was identified further that the convenience of an in-house CME would greatly benefit those professions where it was required as a prerequisite for maintaining licensure [23, 24]. It allowed them to grow their knowledge at their place of work and within work time. In this regard, it would be necessary for the organization to ensure that CME activities obtain the required accreditation to fulfil this need. Accrediting courses would serve as an incentive for participation, thus bolstering enthusiasm and longevity of the initiative [25]. Additionally, CMEs improve knowledge of the different clinical trials ongoing at the site, thus enabling ease of cross-study movement and job versatility.

Several participants across all groups felt there was a distinct advantage to learning together. Similar to other studies, participants felt that despite working at the same research site, they were working in silos. On some level, in this study, interprofessional collaborative learning helped participants develop personal relationships, by putting a face to the names of people that they worked with. Studies have shown that accomplishing this, is a step toward genuine friendships that have the potential to compliment professional relationships and workflows [25]. Thus, interprofessional collaborative education can have benefits that extend beyond the dissemination of knowledge. It has the potential to contribute to a positive work environment through improved collegiality, communication, improved sense of job satisfaction and perhaps lend further to job retention.

They felt that they learned from each other and also about each other’s profession. Through interprofessional collaborative learning, role clarity at the site was established. An understanding of professional roles and responsibilities in a diverse professional environment allows professionals to reach a new understanding of the training, skill set, values, and roles of other professionals in the health care team, thus improving patient’s wellbeing. Being aware of the diverse skill sets available at the research site will possibly enable more efficient and more streamlined care to patients in the future. The study revealed that the clinicians who are the more senior medical staff at the site felt that they had a lot to learn from the other professions. In the past, attitudes and stereotypes held by different healthcare professionals have been cited as a barrier to IPE [26, 27]. Medical practitioners are usually perceived as dominant to other professions and are often relied on to lead or make decisions [28]. This class establishment among healthcare professionals often impedes collaboration and teamwork. However, with the advent of interprofessional collaborative education and collaboration, other members of a patient’s medical team, such as nurses, radiologists, social workers, and professionals from any number of other disciplines, are empowered to make recommendations about patient care. Interprofessional collaboration starts with interprofessional collaborative education [29].

Participants from different professions, present with different learning needs, basic knowledge levels, and different approaches to patient care [28]. This feeds into some of the challenges that were experienced by participants in the study. One such challenge was that interprofessional collaborative learning was intimidating. This was perhaps related to the lack of foundational knowledge by individual participants who were thus not confident to express themselves before the other healthcare professionals, for fear of being incorrect. It has previously been recognized that successful IPE models acknowledged differences among professions in advance [25]. In doing so, students could learn about each other’s profession and were taught to expect differences of opinion. In the current study, this was not explicitly done; however, it had emerged that through the various activities and discussions, participants were enlightened about the roles, responsibilities, experiences, and thinking’s of the other professional. Through this process, they developed a greater respect for members of their immediate healthcare team.

Organizational barriers were also amongst the challenges in implementing a successful interprofessional collaborative CME program. This included identifying suitable times and facilities to conduct training, which would not impede the general running of the clinic [15, 30]. This is an essential factor as chronic understaffing, synonymous with the South African healthcare sector, often impedes the ability to adequately train health workers in HIV care, as removing medical staff from mobile clinics for CME adds to the strain on clinical care systems [31]. Time to attend the lecture was cited as one of the main barriers. They felt that dedicated, protected time should be set aside for this and that the company should acknowledge the value of an in-house CME. Internationally some of the common barriers to external CME included lack of time, lack of resources, and cost [30, 32, 33]. In a similar study to ours, researchers found that their CME activity had experienced comparable problems such as CME scheduling and clinic productivity pressures. Leadership support is listed as crucial to the success of this type of initiative [34] as when different professions are unable to spend meaningful time together due to scheduling conflicts, realizing the benefits to interprofessional collaborative learning will remain a challenge [25].

It was noted that the teaching methods employed during the CME were important to how the participants learnt. For IPE to be appealing, meaningful and relevant to learners, it must be authentic [35]. Active learning, through the use of problem-based learning and cases, facilitated the best learning, as it allowed for the use of educational scenarios which were relevant to all the participants who work together. It further provided insight into how different healthcare professionals at the research site approached a particular problem, thus extending for greater appreciation of each members contribution at the site. Participants particularly enjoyed working in small groups. Small group problem-based learning is a student-centred teaching activity. Evidence suggests that it promotes interest, critical thinking, communication, transference and retention of knowledge [36]. Due to the diversity of the knowledge backgrounds of the participants, prior readings of the subject material were requested by certain participants. This would ameliorate challenges related to non-familiarity with professional jargon, especially scientific and medical terminologies, acronyms, and pharmacological names [37].

Finally, CME training activities should be undertaken internally. Participants had voiced their interest in taking ownership and driving future CME initiatives. This could potentially boost enthusiasm as it allows priority training needs of a particular clinic to be focused upon, thus increasing the likelihood of attendance.

### Strengths and limitations

The main strength of the study rests in all categories of staff working (clinical and non-clinical) at the NGO site being represented in the interviews, thus ensuring a richer understanding of all perspectives relevant to the study site.

One of the limitations was that the researcher (SC) was a former employee at the NGO and her presence at the FGDs could have influenced the responses of the participants. The other being that the use of a small sample of healthcare professionals in a single research site prevents these findings from providing an accurate representation of the sentiments of all healthcare professionals and limits the applicability of its findings.

## Conclusions

Interprofessional collaborative learning against the backdrop of an NGO environment has seldomly been researched. The mix of clinical and non-clinical staff, all participating collectively in clinical trials, research and patient care requires the implementation of teaching opportunities that would drive the organisational goals of achieving improved workplace quality, efficiency and relationships. The categories that have emerged in this study provide guidance as to how the NGO can use teaching as a tool to achieve these goals. Given that participants attested to collaborative practice being promoted through education, management should capitalise on this opportunity to develop competencies that would de-emphasize individual professional needs and roles and promote team goals and collaboration thus leading to a more patient-centred model of care [38].

Clearly, interprofessional collaborative learning was well received and valued by the participants. The learning initiative served as a refresher as well as providing new knowledge on the subject. It improved capacity and work relationships. It also created a better company culture. However, the advancement of this type of initiative would require much input and preparation from the facilitator to plan and structure a curriculum that meets the varying needs of the staff from the institution. There were logistical challenges related to time and resources to conduct this type of training. This would require company buy-in/investment and recognition from leaders and higher management with regards to time and resources. There were many good suggestions such as formalizing the training, introducing more lectures and pitching each topic at different levels i.e. basic, intermediate or advanced, thus ensuring maximum benefit for all. A final recommendation to strengthen the collaborative approach would be to foster discussions and engage a multidisciplinary team of experts to develop the materials for each session, thus commencing the interprofessional approach even before the group sessions begin.

## Supplementary information

**Additional file 1 S1**. Questionnaire (Demographics). **S2**: FOCUS GROUP DISCUSSION GUIDE. **Table 1** Examples of Content Analysis Coding and Categorization.

## Data Availability

The datasets used and/or analysed during the current study are available from the corresponding author on reasonable request.

## Abbreviations

CME: Continuing Medical Education
IPE: Interprofessional Education
NGO: Non-governmental organisation
WHO: World Health Organization
FGDs: Focus group discussions
HIV: Human Immunodeficiency Virus
TB: Tuberculosis
PrEP: Pre-exposure prophylaxis
MCQ: Multiple choice questions
QC officers: Qualitative control
CPD: Continuing Professional Development
