# Supplementary material for: Interprofessional collaborative learning in the workplace: a qualitative study at a non-governmental organisation in Durban, South Africa
Source: BMC Med Educ. 2020 Oct 6;20:346. doi: 10.1186/s12909-020-02264-5 (PMC7541280; doi:10.1186/s12909-020-02264-5)
Supplement: Supplementary file 1 — Additional file 1 S1. Questionnaire (Demographics). S2: FOCUS GROUP DISCUSSION GUIDE. Table 1 Examples of Content Analysis Coding and Categorization. [file 12909_2020_2264_MOESM1_ESM.docx]

**Interprofessional Collaborative Learning in the workplace: A Qualitative Study at a Non-Governmental Organisation in Durban, South Africa**

**Supplementary information**

**Sarentha Chetty^1^, Varsha Bangalee^1^, Petra Brysiewicz^2^**

^1^Discipline of Pharmaceutical Sciences, School of Health Sciences, Westville Campus, University of KwaZulu-Natal, Durban, South Africa

^2^School of Nursing & Public Health, College of Health Sciences, University of KwaZulu-Natal, Durban, South Africa

*Corresponding author: chettys4@ukzn.ac.za*

**S1**

**Questionnaire (Demographics)**

Male Female

Age

20 – 30 30-40 over 40

Highest level of education ___________________________________________

What is your profession? ____________________________________________

Qualifications: _____________________________________________________

What is your job title? ______________________________________________

Where do you work? _______________________________________________

Treatment Prevention Other

If other please specify ___________________

Please tick (√) the number of years of experience working in HIV and TB

| <1 yrs. |  |
| --- | --- |
| 2- 5 yrs. |  |
| 5 -10 yrs. |  |
| > 10yrs |  |

Please tick (√) the lectures that you attended?

| 1. HIV Introduction |  |
| --- | --- |
| 1. HIV treatments |  |
| 1. HIV Standard treatment guidelines (STG) |  |
| 1. HIV Drug resistance |  |
| 1. Gender Issues: Why are women more vulnerable to contracting HIV and PrEP |  |
| 1. Vaccines |  |
| 1. The role of nutrition and food hygiene in HIV |  |

**S2: FOCUS GROUP DISCUSSION GUIDE**

**Format:**

- Welcome and introduction of the researcher and participants
- The topic interprofessional learning in the workplace – will be explained
- Guidelines of how the focus group will be conducted will be explained to participants

**Questions:**

1. ***What are your perceptions of attending this interprofessional HIV CME activity?***

Probes:

1. How do you feel about attending a CME activity with different healthcare professionals and allied staff?
2. ***What are the advantages of attending this interprofessional HIV CME activity?***

Probes:

1. Did it promote learning? If so, how?
2. What did you think were some of the advantages of attending the CME activity with staff from the different clinics (treatment and prevention) and across the different studies?
3. ***What are the challenges/disadvantages to attending this interprofessional HIV CME activity?***

Probes:

1. What did you think were some of the disadvantages of attending the CME activity with different professionals and support staff?
2. What did you think were some of the disadvantages of attending the CME activity with staff from different clinics (treatment and prevention) and across the different studies?
3. ***What are the possible suggestions for improvement of the CME activity?***

Probes:

Any ways to make it more effective, more interprofessional

**S3**

Table 1 Examples of Content Analysis Coding and Categorization.

| Participant | Meaning unit | Condensation | Code | Categories |
| --- | --- | --- | --- | --- |
| 21 | ..everything is changing so quickly we need to learn the newer ways the better ways of doing things.. | ..everything is changing so quickly | Knowledge update | Learning something new |
| 1 | For my team getting insight into information that we don’t work in on a daily basis. | insight into information | New knowledge | Learning something new |
| 9 | It is also nice because you have different people from different sectors. So you have your pharmacy. Your lab staff, you have counsellors, recruiters & you have input from everybody. | different people from different sectors. | Different people different input | Acquiring from each other |
| 4 | Important to learn from experience and you do get theoretical knowledge. But again experiences also to gain more theoretical knowledge. But then again learning from experiences as opposed to learning just directly even just trying to read because most of us know as well that within medicine a lot of it comes from personal experience. | Important to learn from experience and theoretical knowledge | Experiential learning | Acquiring from each other |
| 13 | So there were a lot of studies that were going through [the NGO] where some of us were not involved and we had no information about. So we used to have people asking us about them only to find that when we say to this group I don’t know or I don’t have information. | there were a lot of studies that were going through [the NGO] where some of us were not involved and we had no information about | Improved company knowledge | Promoting company culture |
| 2 | having these series of lectures and learning and laughing together breaks a lot of ice between the professions and I think that I will feel more comfortable and I think a lot of the other staff will feel more comfortable to discuss any number of issues now because we have that rapport built. | more comfortable to discuss any number of issues now because we have that rapport built. | Team building  Networking | Promoting company culture |
| 5 | staff working for such an organization it should have an understanding across the board | have an understanding across the board | Staff should be knowledgeable | Needing company buy-in |
| 7 | CPD has actually become compulsory now and the employer should be assisting in meeting the CPD requirements and this is one simple way in which this can be met. Really easy way of doing it. It is all in house basically. | employer should be assisting in meeting the CPD requirements  easy way of doing it. It is all in house basically. | In house learning | Needing company buy-in |
| 12 | I liked the case studies quite a lot. Even though it was clinical it made you think out of your own whatever you can do whatever your qualification is. | I liked the case studies quite a lot. Even though it was clinical it made you think out of your own whatever you can do whatever your qualification is. | Active learning | Teaching methods matter |
| 18 | We actually learnt. You know the case studies were really very helpful. And do think for all the categories because some of these things are things we have seen so even now in our own communities we now know how to advise people from what we have learnt. | Actually learnt  case studies were really very helpful  we now know how to advise people from what we have learnt | New knowledge  Active learning  Sharing knowledge | Teaching methods matter |
